# Research on programmatic multi-attribute decision-making problem: An example of bridge pile foundation project in karst area

**DOI:** 10.1371/journal.pone.0295296

**Published:** 2023-12-04

**Authors:** Yixuan Lu, Chunlong Nie, Denghui Zhou, Lingxiao Shi

**Affiliations:** College of Civil Engineering, University of South China, Hengyang, China; Libyan Academy, LIBYA

## Abstract

The selection of construction plans for adverse geological conditions frequently encountered during the construction of bridge pile foundations will have a significant impact on the project’s progress, quality, and cost. There is a need for the optimization of multi-attribute decision-making methods, considering the subjectivity in in weight allocation and the practical implementation obstacles. In this study, an evaluation framework for pile foundation construction schemes in karst areas was established. The directed graph and Bellman-Ford algorithm are employed to improve the Analytic Network Process (ANP) in the systematic structure, thereby calculating the subjective weights of various indicators. Simultaneously, based on the concept of dynamic weighting, a multiple linear regression is introduced for analyzing the weights of similar projects, resulting in the derivation of universal weights for the primary indicators within the evaluation system. The combination weights are subsequently determined through the weighted average of the two types of weights. Finally, the comprehensive scores of alternative schemes are computed using the grey-fuzzy evaluation method to enable decision-making in scheme selection. Cloud model, ELECTRE-II, and VIKOR methodologies were utilized for the comparison of results. Combining with a case study of a bridge project in karst development area in southern China, the findings indicate that the improved ANP method possesses practical applicability and yields effective computational results. The introduction of universal weights serves to ameliorate the inherent subjectivity in weight allocation. The pile foundation quality achieved using the optimal construction plan is classified as Class I, which prove the feasibility of the model.

## 1 Introduction

Decision-making is a crucial means of appropriately aligning construction techniques with environmental conditions, thereby achieving the optimal equilibrium for engineering project objectives. Karst terrain, also known as karst topography, constitutes a geological adversity encountered in foundational construction. In China, the karst region covers an area of 3.443 million square kilometers, constituting 35.93% of the total land area [1]. For large-span bridge engineering projects, the probability of encountering unfavorable geological conditions is significantly elevated [2]. Pile foundations serve as the basis for bridges, and inappropriate construction methods could lead to karst ground subsidence, resulting in significant losses[3–5]. The presence of unfilled cavities can result in various construction-related challenges such as collapse, grout leakage, stuck drilling, and excessive concrete pouring [6]. These issues significantly impact the quality, safety, cost, and timeline of bridge pile foundation construction in karst development areas. Therefore, the selection of an appropriate construction approach is a crucial measure to ensure foundation stability, enhance project quality, and meet construction schedules.

For the challenges faced in the construction of bridge foundation engineering in karst area, a research question is posed. How can decision-makers perform multi-attribute decision-making for construction schemes when confronted with complex karst geological conditions? Additionally, it is natural for management personnel to rely on experiential judgment, which results in considerable waste of costs and time. This is considered a secondary motivation for the study.

This paper will introduce a multi-attribute decision-making model for construction schemes, established an evaluation system based on the shared construction objectives of all projects, and intended for broad applicability across construction schemes in karst area. Simultaneously, the study paid attention to the issues of subjectivity in weight allocation and obstacles encountered in the application of existing decision-making methods, prompting enhancements to conventional approaches.

The rest of the paper is organized as follows, Section 2 summarizes the relevant literature on bridge pile foundation engineering, karst treatment techniques, the choice of construction schemes and methods of multi-attribute decision-making. Section 3 encompasses the core methodologies, commencing with the multi-level assessment criteria for bridge pile foundation construction schemes in karst areas. The steps and advantages of the improved Analytic Network Process (ANP) method are expounded upon, universal weights derived from multiple linear regression are introduced, and the section concludes with the establishment of a Grey-Fuzzy comprehensive evaluation model for decision assessment. In Section 4, the model’s effectiveness is validated through a case analysis and comparison with other decision-making methods. Finally, the results and discussions, as well as the conclusions and limitations, are provided in Sections 6 and 7, respectively.

## 2 Literature review

### 2.1 Bridge pile foundation engineering in karst area

Currently, due to the complexity of karst geological formations, bridge pile foundation engineering has been extensively studied. On one hand, existing research is concentrated on innovative construction processes, investigations into structural mechanical properties, analysis of construction risks, and comprehensive evaluations of technical indicators. For instance, Huang et al. [7] introduced a novel theoretical model for a large-diameter stepped-tapered hollow pile, addressing the issue of instability in the bearing layer due to karst geological conditions. He et al. [8] established models to calculate the side resistance, frictional resistance, and pile-end resistance of rock-socketed piles in the karst area. Xing et al. [9] assessed the impact of various pile driving techniques and grouting methods on the mechanical properties of rock-socketed piles. Ji et al. [10] based on the Yinzhou Lake Bridge project, identified sources of risk within divisional work and conducted risk prioritization. Li et al. [11] constructed evaluation models to assess the construction safety risks of highway bridges. Jin et al. [12] conducted an evaluation of grouting effectiveness in karst areas, considering the construction technology, apparent parameters, observational data from inspection holes and geophysical exploration techniques. On the other hand, prior to construction, risk reduction is achieved through the detection of karst features with the aim of mitigating construction risks. Detection methods encompass ground-penetrating radar (GPR) [13], electrical resistivity tomography (ERT) [14], transient electromagnetic method (TEM) [15], crosshole seismic Computed Tomography (CT) [16] and so on. However, GPR, ERT, and TEM are suitable for karst exploration closer to the surface, and traditional surveying techniques have limitations when it comes to deeper or larger-scale karst features [17]. Hence, research into the prediction of karst features has been developed [18]. For instance, Zhang et al. [19] proposed the construction of an assessment system to predict the state of karst development. This approach involves using fuzzy evaluation methods to provide initial karst predictions before construction, and during construction, combining various geological survey techniques to refine the exploration of karst features.

### 2.2 Karst treatment techniques and scheme decision-making

Due to the complexities associated with karst geological exploration, the karst treatment methods during the pile foundation drilling process have become a technological challenge. LI et al.[20] based on the foundation construction examples of the Zhonglao Railway bridge in karst area, summarized the applicable treatment techniques, selection principles, and control points for different types of karst development. Dong et al. [21] analyzed the bearing capacity of pile foundation characteristics of the pile foundation for the double-layer retaining wall by using numerical simulation verified the feasibility of the double-layer steel casing retaining wall in super-large karst caves. LI et al. [22] putted forward a set of management procedures for karst gushing water treatment, and introduced further-more the precise exploration method, grouting material, construction steps and effectiveness check for the karst gushing water treatment in detail [23].

The literature mentioned above is focused on the exploration of construction techniques. However, in engineering construction, it is essential to consider not only meeting technical requirements but also addressing multiple constraints, including economic factors. For instance, Chen [24] conducted a quantitative comparison of karst area pile foundation construction schemes, considering both project duration and cost aspects. Pan [25] posits that selecting an appropriate bridge construction method is a process of balancing multiple factors, including cost, quality, project duration, safety, and bridge design. For the lean management of construction schemes, Li et al. [26] utilizes a management framework comprising five dimensions: quality, cost, time, safety, and organization during the construction process. The Analytic Network Process (ANP) method is considered highly suitable for evaluating systems that involve complexity, nonlinearity, as well as a mix of qualitative and quantitative indicators. Similarly, considering the complex geological characteristics and construction challenges in karst areas, pile foundation construction schemes can be defined as a Multiple Attribute Decision Making (MADM) problem involving multiple factors, objectives, and criteria.

Some scholars have engaged in discussions regarding decision-making for bridge pile foundation construction schemes. Zheng [27] focused on the decision-making for bridge construction schemes across rivers. They established an evaluation system based on four aspects: technical performance, economic benefits, implementation effectiveness, and environmental effects. The weightings were determined through expert rating, and the selection of the scheme was carried out using fuzzy evaluation. Yu and Pan [28] developed an evaluation model using the Analytic Hierarchy Process (AHP), facilitating the comparative analysis of pile foundation construction schemes for mountainous area renovation and expansion projects. Among these methods, AHP is better suited for linear evaluation systems. It exhibits a high degree of subjectivity, which can lead to potential biases in decision-making due to individual differences. Wu [29] formulated a fuzzy comprehensive evaluation model for comparing bridge pile foundation construction schemes near the operating railway lines in the western mountainous regions. They applied the Maximum Deviation Method for objective weighting, intending to mitigate the influence of subjective factors on weight allocation. However, the decision-making methods in these studies are relatively simplistic, and there is room for further enhancement in modeling approaches. The limited research volume in the field of pile foundation engineering decision-making in karst areas has led to a knowledge gap. Particularly, in managing the challenges of multi-attribute decision-making, issues persist regarding incomplete evaluation systems, one-sided and inefficient decision-making methods. Therefore, it is necessary to establish a scientific, efficient, and practical decision-making model.

### 2.3 Multiple attribute decision making

Compared to Multi-Objective Decision Making (MODM) methods, Multiple Attribute Decision Making (MADM) is better suited for decisions involving a limited set of alternative schemes [30]. In order to address intricate decision-making challenges, Multiple Attribute Decision Making (MADM) methods have found extensive applications across various domains, including engineering decision support systems [31], finance [32], industrial design [33], and other fields. For instance, Więckowski [34] posited that supplier evaluation constitutes a Multi-Criteria Decision Analysis (MCDA) problem. Using Triangular Fuzzy Numbers in combination with five MCDA methods, suppliers are ranked and selected, with the robustness of the results examined through sensitivity analysis. Jagtap [35] innovatively improved the Simos’ method for weight calculation in the issue of selecting non-traditional machining processes. The results demonstrated greater stability in comparison to the AHP method. Specially, a dynamic directed graph was employed to visualize the preferences among alternative options. The decision-making process was conducted using the M-Polar Fuzzy Set ELECTRE-I method. Ors [36] addressed the issue of selecting bridge pier construction schemes and designed an AHP-TOPSIS model for choosing the optimal construction technique.

Additionally, a combination of weighted integrated evaluation was proposed to overcome the limitations of a single weight. Zheng et al. [37] considered the comparative intensity and conflicts among evaluation values, using the CRITIC method for objective weight calculation and minimum discrimination information for weight aggregation. Xu [38] considered the information content contained in each indicator and employed the entropy weight method to calculate objective weights, which is particularly suitable for quantitative metrics. However, objective weighting is calculated solely based on subjective score values, which has certain limitations.

Then, the process of selecting a solution requires both qualitative and quantitative analyses, and many pieces of information exhibit fuzziness and incompleteness. Fuzzy theory [39] represents one of the widely applied methodologies in such cases. Mahmood Shafiee [40] considered wind energy site selection as a complex decision problem with a high degree of uncertainty, and proposed the FANP-TOSIS model to evaluate potential sites for wind power development. In response to the challenges in selecting an optimal blasting plan for a certain bauxite deposit with significant dimensions, Wu [41] et al. integrated Analytic Network Process (ANP), Fuzzy Theory, and TOPSIS theory to make the blasting plan selection.

Xin et al. [42] based on the grey theory, calculated the evaluation grey category for each rating value using the whitenization weight function. This effectively illustrates the degree of membership of each indicator to different levels, addressing the issue of quantitative assessment in the bridge inspection system. In the multi-objective decision-making problem for the selection of railway route schemes, Li et al. [43] replaced the evaluation of qualitative indicators with interval fuzzy numbers and transformed them into cloud model features. This approach effectively addresses the limitations related to the fuzziness and randomness in the evaluation method. Hence, the paramount consideration in addressing multi-attribute decision-making challenges is to ascertain the optimal methodologies for enhancing the efficiency and applicability of decision processes.

### 2.4 Comparison of MADM methods

Weight calculation methods can be primarily categorized into two major classes: subjective weighting, which determines weights through comprehensive consultation-based scoring, and objective weighting, which calculates weights based on interrelationships or variations among indicator values. Prominent integrated decision-making methodologies encompass TOPSIS, cloud model, GRA, ELECTRE, VIKOR, among others. Drawing from the existing research, these approaches will be synthetically elucidated and compared, as shown in **Table 1**.

**Table 1:** Comparison of methodologies in scheme selection.

Methodology	Attribute	Limitation	Reference
AHP	The evaluation system is decomposed into a hierarchical structure, and subjective weights are calculated through pairwise comparisons.	The lack of consideration for interrelationships between indicators results in a situation where decision-makers may face difficulties in making judgments when confronted with a substantial number of indicators.	[10,25,35,44,45]
Ordering Relation Analysis method (G1)	Ordinal relationships and preference scores are directly supplied, negating the requirement for consistency checks.		[46,47]
ANP	A network hierarchy is established, taking into consideration the interrelationships between indicators.	A highly complex calculation process and a highly challenging implementation.	[26,40,48]
Entropy Weight Method (EWM)	The information entropy of indicators is employed to reflect the discriminative capability towards the evaluated subjects.	The quality of sample data plays a decisive role, and outliers cannot be accommodated.	[17,37,44,45]
The Criteria Importance Through Intercriteria Correlation (CRITIC)	The variability and conflict of the indicators are employed to calculate the objective weights.		[40,41,45]
GRA	The correlation between each alternative and the optimal solution is computed.	The subjective selection of the optimal solution and the parameter can lead to variations in the results.	[42,44,45]
TOPSIS	The distances between alternative solutions and the best and worst solutions are calculated	Errors may exist in the calculation of distances, and uncertainty information cannot be handled.	[36,44,45]
VIKOR	Solutions that exhibit the closest approximation to the ideal solution are determined through an analysis of S,R,Q.	The variation in results is attributed to the configuration of the threshold.	[36,45]
ELECTRE (Elimination and Choice Expressing Reality)	Subsequent to threshold configuration, the Concordance Matrix, Discordance Matrix, and Dominance Matrix is conducted to calculate the ranking process.		[28,29]
Fuzzy Comprehensive Evaluation	The utilization of fuzzy mathematical theory is employed to assess the membership degrees between the indicator set and the evaluation set.	The selection of the membership function exhibits an inherent randomness.	[19,26]
Cloud Model	The conversion between qualitative concepts and quantitative values is enabled.	Subjective assessments have a substantial impact, suitable for ordinal evaluations.	[11,46]

The literature review presented above illustrates that the construction technology for bridge pile foundations in karst areas and the exploration techniques have been consistently developing and making breakthroughs. However, there has been limited exploration regarding the multi-attribute decision-making for pile foundation construction schemes. While the methodological framework for multi-attribute decision-making problems is relatively comprehensive, each approach still exhibits its own inherent limitations. This study contributes to the fusion of theoretical and practical aspects concerning project management issues in the field of bridge pile foundation construction. It is crucial to emphasize that, with the aim of promoting sustainability, in research representing the technical disciplines, the importance lies not only in innovative technical methods but also in the paramount practicality of research outcomes [49]. Theoretical research can provide a foundation for the implementation of new technologies, and it is also essential for construction preparations to include assessments of costs, quality, and safety. Therefore, research on scheme decision-making is meaningful. In practical engineering construction, traditional karst treatment methods are determined by a single influencing factor. The decision-making process is characterized by strong subjectivity, expert reliance, and difficulties in harmonizing diverse opinions. The constructed evaluation criteria system still faces issues related to comprehensiveness and limited applicability. To address these challenges, the adoption of appropriate decision models becomes essential.

Then, a novel approach is proposed to study the decision-making process for pile foundation construction schemes in karst areas. Firstly, an evaluation criteria system for construction schemes is constructed, taking into account technical, economic, quality, and environmental aspects. An important point to consider is the utilization of directed graphs within the system structure model and the Bellman-Ford algorithm in the expert rating phase of the Analytic Network Process (ANP). In the form of distributing survey questionnaires, aim to seek subjective ratings from domain experts for the indicators, and then input the obtained ratings into the Super Decision software to derive the indicator weights. Drawing on the concept of dynamic weighting, the introduction of universal weights serves as objective weights. Subsequently, The data from relevant research studies is analyzed. Multiple linear regression is applied to these data, and the correlation coefficients among four indicators within similar projects are calculated. This enables the establishment of linear relationships between the decision-making and control layer indicators, which are then weighted to determine their subjective importance at the control level. Based on the concept of dynamic weights, it is employed as a universal weight and integrated with the control layer indicators’ weights to constitute composite weights. Finally, by integrating grey fuzzy evaluation, the scores for each alternative scheme are calculated, which facilitates the decision-making process for the construction plan. A case study of a railway bridge project in southern China is conducted to validate the feasibility of this research methodology.

## 3 Methodology

### 3.1 Construction of the evaluation indicator system

Based on the characteristics of pile foundation construction in karst regions and engineering practices, and with reference to similar literature [50–63], four primary indicators and sixteen secondary indicators were constructed.

Taking into account the four major management elements at the construction site, the technical criteria, economic criteria, working environment, and safety and quality were used as primary evaluation indicators. Following comprehensive, objective, and scientific principles, the assessment of the construction schemes’ merits was conducted.

Focusing on the 16 secondary indicators, decision analysis was conducted. Considering the characteristics of these indicators, U_13_, U_31_, U_32_, and U_35_ are quantitative indicators, while the remaining indicators are qualitative.

The evaluation index system is presented in **Table 2**.

**Table 2:** The evaluation index system.

Control layer	Network layer	Description of indicators
Technical criterion U_1_	Selection of construction machine U_11_	Drillability of geological conditions
		Dynamic parameters of machinery
		Flow plastic soil conditions
		Drilling speed
		Frequency of machine maintenance
	Sequence of construction process U_12_	Preparation process before construction
		Drilling construction process
		Karst construction process
		Process of following steel casing
	Time limit of construction U_13_	Combining the realities of engineering and the k-means algorithm
	The difficulty of construction U_14_	The length of the piles
		Geological condition
		Construction organization
		Restrictions of construction site
Economic criterion U_2_	The construction cost of karst cave U_21_	Volume of concrete over-irrigation
		Volume of flag stone and clay
		Length of lower steel
		Volume of mud
	Quality repair cost U_22_	Cost of geologic investigation
		Cost of inspection of pile body
		Construction platform collapse
		Cost of labour
		Cost of material
	Construction cost U_23_	Labour cost
		Cost of material
		Cost of machine
		Measure expenses
		Cost of other project
Construction environment U_3_	Thickness of sand layer U_31_	The data is extracted from geotechnical investigation records, and the classification is determined based on literature sources and k-means algorithm
	Thickness of the first rock layer U_32_	
	Filling degree of karst cave U_33_	
	Weathering degree of rock layer U_34_	
	The maximum height of karst cavity U_35_	
Quality and safety U_4_	Construction management system U_41_	Personnel management
		Responsibility in construction
		Safety inspection
		Safety protection measure
		Construction site management
	Construction technology control measures U_42_	Preparation measures before construction
		Pile foundation design
		Hole drilling
		Indicators of Technical specification
	Material management measures U_43_	Management system of acceptance materials
		Management system for rental machine
		Management system of material placement
		Management system of material recovery
		Management system of material arrangement plan
	Emergency response plans U_44_	Emergency plan of accidents
		Emergency plan of casualties
		Emergency plan of mechanical failure
		Emergency plan of operation platform collapse
		Emergency plan of construction process accident

### 3.2 Improved Analytic Network Process (ANP) method

The Analytic Network Process (ANP) [64], proposed by Professor Saaty as an extension of the Analytic Hierarchy Process (AHP), is specifically designed to accommodate non-independent hierarchical structures. This approach takes into consideration the interrelationships among various factors, employing nonlinear structures instead of linear structures and accounting for the dominance relationships among different hierarchical elements.

During the construction process, there exist interdependencies and inseparable relationships among the technical, economic, environmental, and quality criteria. Therefore, the adoption of the ANP method for analysis is more appropriate. The ANP comprises a control layer and a network layer. It involves establishing network relationships among different criteria to assess the pairwise comparisons between indicators and construct an evaluation matrix. By calculating the weighted hypermatrix and the limit hypermatrix, which consider both relative preferences and importance degrees, the weight values for each second-level indicator are determined.

The comparative judgment matrix used in the ANP is a positive reciprocal matrix that possesses transitivity. Therefore, in the evaluation process, it is necessary to examine the consistency of the judgment matrix through the Consistency Ratio (CR). If the CR value is greater than 0.1, it is considered that the judgment matrix satisfies consistency, and the test is passed.

The comparison and selection of pile foundation construction schemes are complex decision problems involving multiple attributes and criteria. During the decision-making process, when experts are invited to participate in group decision-making, the following issues typically arise:

1. Different experts provide similar or conflicting evaluation opinions, and after collecting the rating data, the decision matrix may not even pass the consistency test, leading to inefficiencies in the indicator evaluation process.
2. When there is a large number of experts, there are certain difficulties in achieving consensus and integration of the final scoring values.
3. The conventional ANP method requires experts to pairwise compare indicators, and it becomes challenging for experts to intuitively assess the relative importance of factors from an overall perspective.

To address the above-mentioned issues, directed graphs and the Bellman-Ford algorithm are introduced into the system structure to improve the ANP method. A directed graph, originating from graph theory, consists of nodes and directed edges, where nodes represent elements, and directed edges represent the influence relationships among these elements. HUANG [65] based on the Interpretative Structural Modeling Method to elucidate the rules for describing the relationships among elements using a directed graph. They also illustrated the process of transforming a directed graph into an adjacency matrix, wherein, if element A exerts an influence on element B, the corresponding element in the adjacency matrix is denoted as 1. Hence, the directed graph has become an effective tool for visualizing connections among elements. GAO [66] used directed graphs to represent the cause-and-effect relationships between explicit and implicit factors in the enterprise environmental assessment information system, with the direction of the edges indicating positive or negative influences and reachability relationships. Bellman-Ford algorithm [67] originates from the computational method for the shortest path in graph theory. In comparison to the Dijkstra algorithm, it has the advantage of being able to handle negative weights.

The introduction of directed graphs primarily aims to visualize the evaluation objects, enabling experts to have a holistic perspective and make intuitive judgments of the evaluation objects, aligning with the central philosophy of the ANP method. Following the principles of G1, experts are required to convey the importance of evaluation criteria in an ordered relationship from left to right, ensuring the consistency of the judgment matrix. The numerical values along the edges directly reflect the significant relationships between adjacent criteria. In order to maintain consistency with the 1–9 scale scoring method, the improvement is made by adopting a five-segment scoring scale, thus providing a clear quantification of the importance values for pairwise comparisons of factors. Subsequently, BF algorithm performs pairwise weight calculations between the criteria, achieving the transformation from a directed graph to a judgment matrix, serving as a bridge between graphical and matrix representations.

Step1: Inviting experts to rank the importance of factors in the network layer and criterion layer, denoted as $a_{ij_{1}}>a_{ij_{2}}$. Quantifying the importance values between pairwise comparisons of metrics using the set {0.5, 1, 1.5, 2, 2.5} corresponding to progressively increasing levels of importance.


$$\{\begin{array}{c} a_{ij_{1}}=a_{ij_{2}}+\Delta r_{j_{1}j_{2}}\\ 0<\sum_{m=1}^{n}a_{ij_{m}}<9\\ a_{ji}=1/a_{ij} \end{array}$$ (1)

Where $a_{ij_{k}}$ is the rating value of the j_k_ indicator in the i-th layer; $\Delta r_{j_{1}j_{2}}$ is the importance value between the two indicators.

Step2: Converting different evaluation orders into ascending sequences, where the assignment of values between elements is determined based on the cumulative summation operation using the Bellman-Ford algorithm.

**Fig 1:**
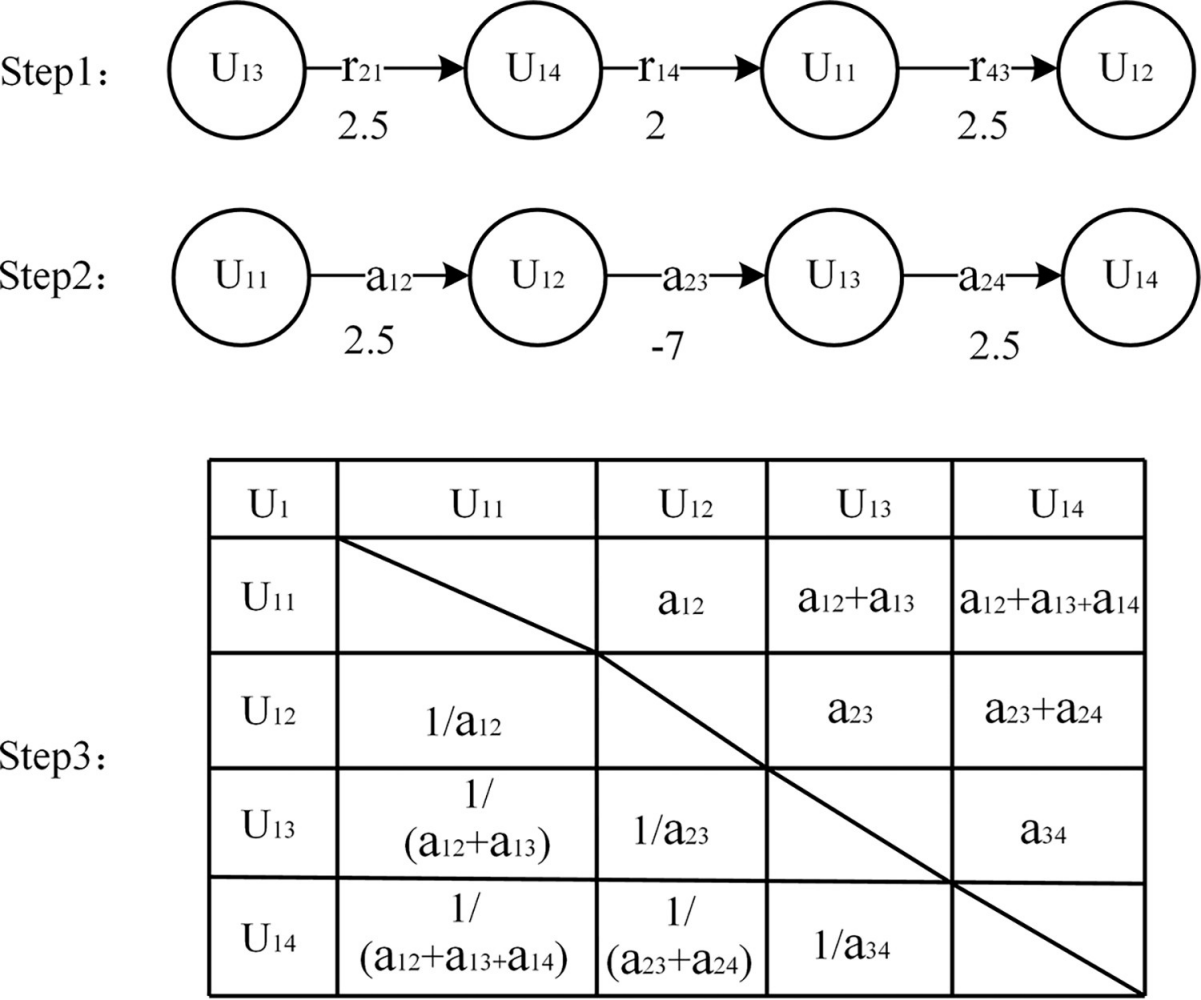
Expert scoring flow chart.

Step3: For a single expert, the judgment matrix can be directly obtained from the ascending evaluations. In the case of multiple experts, multiple sets of ascending scores are collected, and their average values are rounded to obtain the judgment matrix. The evaluation process for a single expert is illustrated in **Fig 1**.

Step4: Calculate the Consistency Ratio (CR) for each judgment matrix. If CR < 0.1, it passes the consistency test.

Step5: Utilizing the Super Decision software, the hypermatrix matrix and limit matrix were calculated to obtain the indicator weights.

### 3.3 Prediction of universal weights based on MLR

Weights are typically computed by experts around a specific instance or evaluation system, reflecting a static perspective. Jiang [68] recognized often disregarded dynamic changes within indicators and introduced a dynamically weighted grey optimization model, suggesting that the weight assigned to indicators is dependent on their amplitude of change across distinct categories. Wang [69] employed historical data pertaining to aqueduct engineering as a universal reference weight, thereby facilitating the dynamization of evaluation indicator weights. Li [70] He conducted a frequency analysis of the occurrences of risk impact factors to compute universal weights, emphasizing the reference value of historical data from similar construction projects. Therefore, the introduction of universal weights is conducive to reflecting the objectivity and dynamic changes of indicator weights.

#### 3.3.1 Multivariate linear regression

Regression analysis is a commonly used method in mathematical statistics, based on quantitative analysis of the relationship between the dependent variable and independent variables. It establishes a mathematical expression describing the mapping relationship between variables through the observation of data and the existing variables, thereby enabling the estimation and prediction of unknown data. Multiple regression analysis is a method of establishing a regression equation with multiple independent variables based on their observed values in relation to the dependent variable. It involves testing and analyzing the significance of the combined linear effects of each independent variable on the dependent variable, in order to assess the quantitative relationship between multiple independent variables and the dependent variable [71]. Multiple Linear Regression (MLR) is extensively employed in research studies exploring the correlations among different variables. For instance, LI et al. [72] employed the multiple linear regression method to obtain the relationship among the critical distance from the pile bottom to the top of the cave, the size of the cave, and the design load of the single pile. Their findings revealed highly significant relationships among these variables. Yildizel [73] probed the correlations between four factors, namely fiber content, calcium carbonate content, sand blasting, and polishing properties of the specimens, and the British Pendulum Number of the glass fiber-reinforced tiling materials. Assessment and prediction were carried out using the Adaptive Artificial Neural Network (ANN) and MLR method. Erzin [74] utilized Multiple Regression Analysis to investigate the correlation between nine factors and the California Bearing Ratio (CBR) value of the soils. The factor with the highest correlation coefficient demonstrated a strong relationship. Evidently, ANN and MLR are both commonly employed methods for evaluation and prediction. When dealing with larger sample sizes, the ANN model exhibits superior predictive performance, with a higher R2 for the test dataset compared to the training dataset [75]. Given the limited availability of similar studies, this paper utilized Multiple Linear Regression (MLR) for data analysis to further achieving the prediction of universal weights.

#### 3.3.2 Prediction of universal weights

In research concerning decision-making, it is common to establish an indicator system to assess the strengths and weaknesses of various schemes. The calculated weights for different indicators reflect the relative importance of each criterion in the project scheme evaluation. Moreover, in the context of research related to the evaluation of similar schemes, the weight values of criteria for different projects hold certain reference significance.

The control layer indicators also serve as common objectives for all engineering constructions, rendering the evaluation system comparable. Utilizing the assessments of these indicators’ significance by other scholars as references, the objective nature of weight determination is maximally pursued.

**Fig 2:**
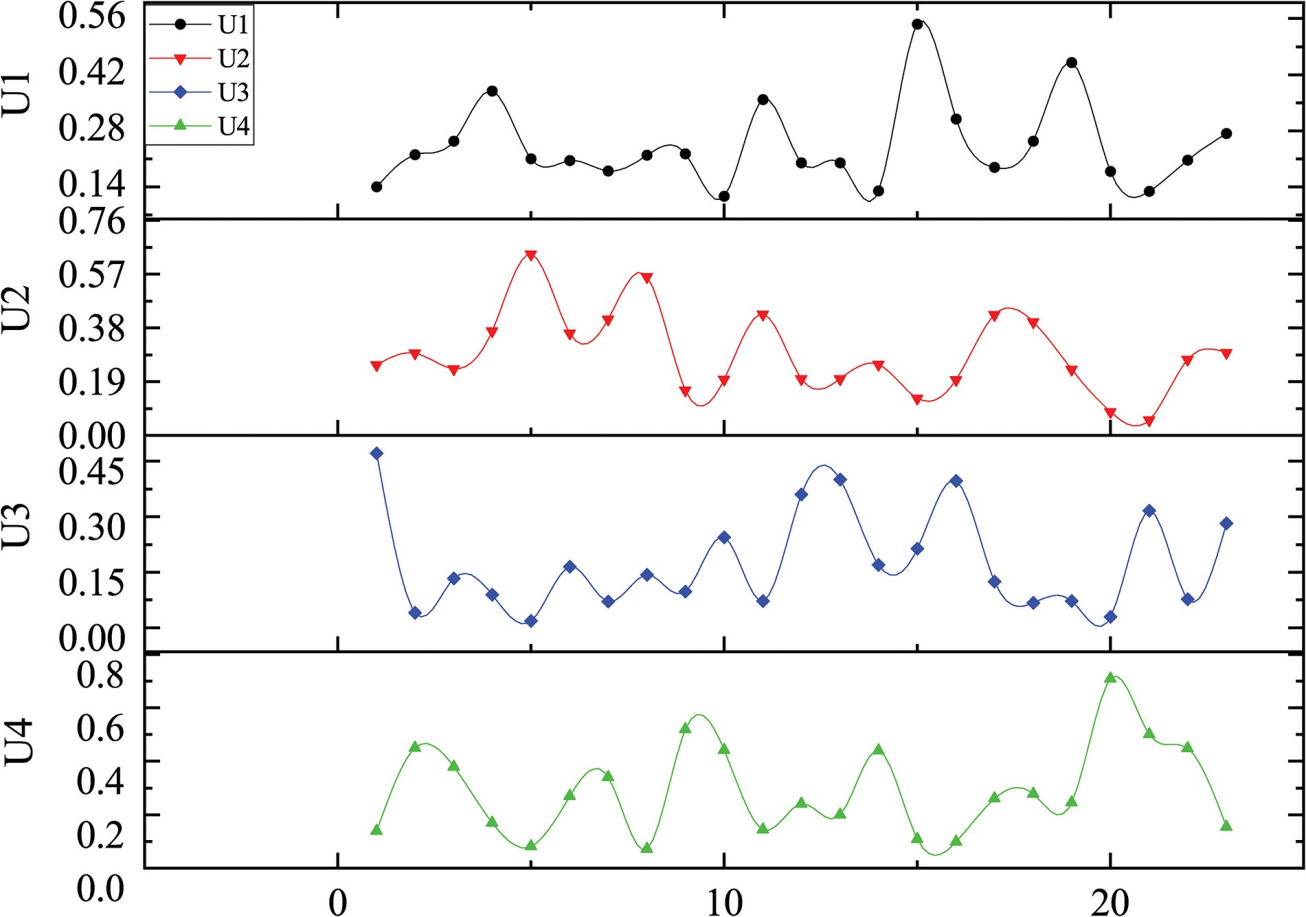
Weights of sample data.

Data acquisition entailed a systematic literature search, utilizing key terms such as "construction scheme selection," "construction scheme optimization," and "scheme comparison" to identify relevant papers in the same domain. Twenty-three papers [27–29,76–95] were gathered using the commonality of control layer indicators as the filtering criterion. Weight data was organized, analyzed, and then merged with criteria of the same type, resulting in the formation of the sample dataset, as shown in **Fig 2**. Subsequently, by utilizing multiple linear regression, the regression equations between different criteria are calculated to establish the linear relationship between decision-making schemes and the four evaluation criteria, obtained the universal weights for similar projects, thus enabling the prediction of weights for this project. The calculation steps are as follows:

Step1: Establishing the overall regression linear equation.


$$y_{i}^{t}=\beta_{0}+\beta_{1}^{t}x_{i1}^{t}+\cdots +\beta_{p}^{t}x_{ip}^{t}+\varepsilon_{i}^{t}\;i=1,2,\cdots ,n$$ (2)

Where $y_{i}{}^{t}、x_{i}{}^{t}$ are respectively the dependent variable and the independent variable., *β*_0_ is the parameter. $\varepsilon_{i}^{t}$ is the error term, ε~N(0,σ^2^).


$$y^{t}={\left(\begin{array}{c} y_{1}{}^{t}\\ y_{2}{}^{t}\\ \vdots \\ y_{n}{}^{t} \end{array}\right)}_{n\times 1}(t=x_{t},x_{c},x_{e},x_{q})$$ (3)

Where *X*_*t*_,*X*_*e*_,*X*_*c*_,*X*_*q*_ are respectively technical criterion, economic criterion, construction environment and quality and safety criterion.


$$\beta^{t}={\left(\begin{array}{c} \beta_{0}{}^{t}\\ \beta_{1}{}^{t}\\ \vdots \\ \beta_{p}{}^{t} \end{array}\right)}_{(p+1)\times 1}X^{t}={\left(\begin{array}{ccccc} 1 & x_{11}{}^{t} & x_{12}{}^{t} & \cdots & x_{1p}{}^{t}\\ 1 & x_{21}{}^{t} & x_{22}{}^{t} & \cdots & x_{2p}{}^{t}\\ 1 & \vdots & \vdots & \vdots & \vdots \\ 1 & x_{n1}{}^{t} & x_{n2}{}^{t} & \cdots & x_{np}{}^{t} \end{array}\right)}_{n\times (p+1)}$$ (4)

Then t cross product matrix of the variable augmented matrix be shown as:

$$V^{t}=(\begin{array}{cc} (X^{t})'X^{t} & (X^{t})'y^{t}\\ (y^{t})'X^{t} & (y^{t})'y^{t} \end{array})=(\begin{array}{cc} V_{11}^{t} & V_{12}^{t}\\ V_{21}^{t} & V_{22}^{t} \end{array})$$ (5)

Step2: Calculation of the regression coefficients.


$$\hat{\beta }={\left(V_{11}^{t}\right)}^{-1}V_{12}^{t}$$ (6)

Step3: Joint significance test of the regression equation.


$$\begin{array}{l} SST=\sum_{i=1}^{n}{\left(y_{i}-\bar{y}\right)}^{2}\\ SSE=\sum_{i=1}^{n}{\left(y_{i}-{\overset{⌢}{y}}_{i}\right)}^{2}\\ R_{adjusted}{}^{2}=1-\frac{SSE}{SST} \end{array}$$ (7)

Where SST is Sum of total difference squares; SSE is residual sum of squares; $\bar{y}$ is the average value of y; $\hat{y}$ is the fitting value of the i-th sample; y_*i*_ is the regression value of the i-th sample.

By individually setting the technical criterion (Xi), economic criterion (Xc), construction environment (Xe), and quality and safety criterion (Xq) as dependent variables, and the remaining three criteria as independent variables. Based on multiple linear regression, identify the correlation between the dependent variable and independent variables, and calculate the four regression equations. The regression coefficients are then used to assign weights to different criteria.

The results of the joint significance test are presented in **Table 3**.

**Table 3:** Parameters of joint significance test.

Equation	F	Adjusted R^2^	prob>F
1	870.48	0.9916	0
2	808.35	0.996	0
3	1444.77	0.9949	0
4	2314.89	0.9968	0

Clearly, p < 0.05, indicating that at a 95% confidence level, the regression coefficients of the four equations are significantly different from zero. Therefore, the null hypothesis can be rejected, indicating the existence of a correlation between the dependent variable and independent variables.

By combining the four regression coefficient equations, the correlation coefficient equations for four independent variables can be solved based on the sample data from the multi-attribute decision-making research of similar schemes. These coefficients can be considered as the objective weight of the four criteria based on the sample data. The equation is as follows:

$$Y=0.2421x_{t}+0.2885x_{c}+0.1758x_{e}+0.2908x_{q}$$ (8)

### 3.4 Grey-fuzzy comprehensive decision model

Fuzzy comprehensive evaluation can comprehensively and integratively assess all factors of the evaluated object based on the principle of maximum membership degree, thereby determining the membership degree level of the evaluated object [96].Based on expert ratings, the introduction of whitenization weight functions is utilized to determine the membership degrees of evaluation values with respect to the grades. This approach leverages the advantages of membership degree to describe the boundaries of scheme evaluation grades, achieving the integration of qualitative and quantitative research and resolving the qualitative and quantitative issues of multi-attribute evaluation. It overcomes the subjective bias of individual decision-making and enhances the effectiveness and practicality of group decision-making.

#### 3.4.1 Determination of the indicators sets and the evaluation sets

Investigating engineering instances of pile foundation construction projects in karst areas, establishing four criteria as the control layer, and delineating 16 evaluation factor indicators as the evaluation indicator set U = {u1, u2, …, u16}. The evaluation set V = {Ⅰ, Ⅱ, Ⅲ, Ⅳ, Ⅴ} is divided into five levels, corresponding to excellent, good, moderate, pass, and fail. This reflects the quality of the pending construction schemes and establishes the score value ranges for each level [97], as shown in **Table 4**.

**Table 4:** Score of evaluation level.

Level	Ⅰ	Ⅱ	Ⅲ	Ⅳ	Ⅴ
Score	90	80	70	60	50

#### 3.4.2 Construction of the evaluation matrix

Through the utilization of the Delphi method and questionnaire surveys, expert evaluations of the proposals were collected, thus constituting an assessment matrix.

#### 3.4.3 Construction of the triangular whitenization weight function

Due to the subjectivity of the ratings provided by management personnel, determining the rating levels is an urgent issue that needs to be addressed. Typically, in fuzzy comprehensive evaluation, it is difficult to determine the membership functions. Therefore, the concept of gray numbers is adopted to assess the membership of rating values to different gray classes.

**Fig 3:**
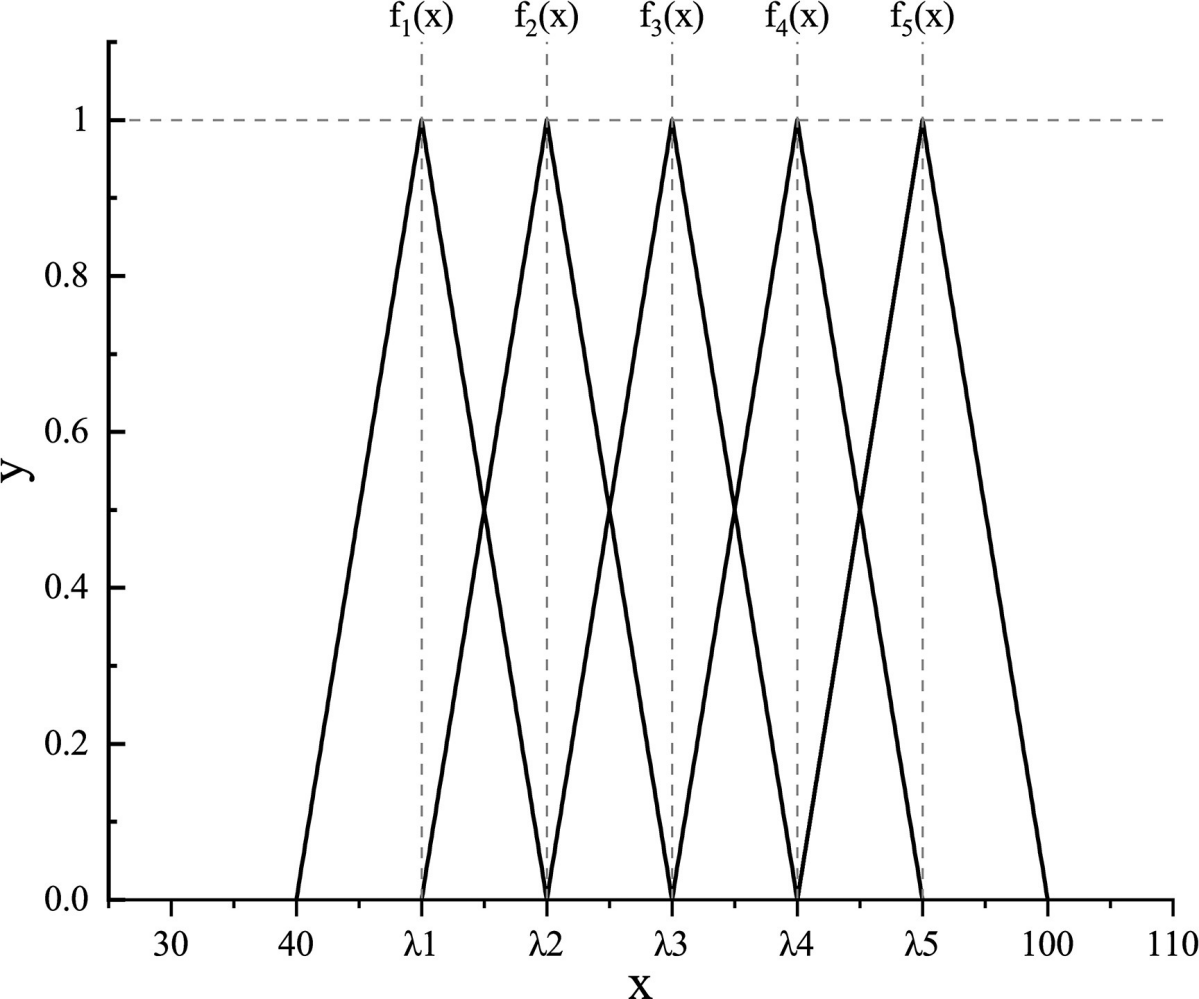
Diagram of triangular whitenization weight function.

The utilization of the central point whitenization weight function helps to avoid the issue of multiple gray classes covered by the same rating value, thereby preventing ambiguity in membership to different levels [98]. The calculation steps are as follows: (1) Assuming there are n candidate schemes, the value range of the 16 evaluation criteria is divided into five gray classes. (2) Let the rating value be defined as the centroid λk of the k-th gray class. The most probable range of rating values for the k-th gray class is [λ_k-1_, λ_k+1_], k = 1,2,…,5. The left endpoint of the first gray class and the right endpoint of the fifth gray class are obtained by extending the rating value range to the left and right, respectively. Subsequently, by connecting the starting point of class k-1 with the center point of class k, the triangular whitenization weight function fj can be constructed. The expression is shown in **Table 5**, and the function graph is depicted in **Fig 3**. (3) Calculate the membership degree Rij of each rating value dij belonging to class k.

**Table 5:** Expression of triangular whitenization weight function.

Level	Whitenization Weight Function
Ⅰ (V_1_ = 90)	$f_{1}(d_{ij})=\{\begin{array}{c} 0,d_{ij}\notin [80,100]\\ \frac{d_{ij}-80}{90-80},d_{ij}\in [80,90)\\ \frac{100-d_{ij}}{100-90},d_{ij}\in [90,100] \end{array}$
Ⅱ (V_2_ = 80)	$f_{2}(d_{ij})=\{\begin{array}{c} 0,d_{ij}\notin [70,90]\\ \frac{d_{ij}-70}{80-70},d_{ij}\in [70,80)\\ \frac{90-d_{ij}}{90-80},d_{ij}\in [80,90] \end{array}$
Ⅲ (V_3_ = 70)	$f_{3}(d_{ij})=\{\begin{array}{c} 0,d_{ij}\notin [60,80]\\ \frac{d_{ij}-60}{70-60},d_{ij}\in [60,70)\\ \frac{80-d_{ij}}{80-70},d_{ij}\in [70,80] \end{array}$
Ⅳ (V_4_ = 60)	$f_{4}(d_{ij})=\{\begin{array}{c} 0,d_{ij}\notin [50,70]\\ \frac{d_{ij}-50}{60-50},d_{ij}\in [50,60)\\ \frac{70-d_{ij}}{70-60},d_{ij}\in [60,70] \end{array}$
Ⅴ (V_5_ = 50)	$f_{5}(d_{ij})=\{\begin{array}{c} 0,d_{ij}\notin [40,60]\\ \frac{d_{ij}-40}{50-40},d_{ij}\in [40,50)\\ \frac{60-d_{ij}}{60-50},d_{ij}\in [50,60] \end{array}$

#### 3.4.4 The comprehensive evaluation result

The second-level indicator comprehensive evaluation set bj is calculated using the fuzzy operator M(·,+) according to the following formula:

$$b_{j}=\sum_{i=1}^{16}\omega_{subject}{}^{j}•r_{ij},j=1,\cdots ,5$$ (9)

The comprehensive evaluation set Bj of the control layer indicators is calculated using the following formula:

$$B_{j}=\sum_{i=1}^{4}\omega_{combined}b_{ij},j=1,\cdots ,5$$ (10)

The comprehensive scores Z for each candidate scheme are calculated according to the following formula, based on which comprehensive ranking can be conducted.


$$Z=\sum_{i=1}^{5}B_{j}V_{i}$$ (11)

### 4 Empirical analysis

#### 4.1 Engineering situations

The bridge project is located in the southern part of China, with a total length of approximately 3600 meters. The pile foundation lengths range from 13.5 to 74.5 meters, with a total quantity of about 1500 piles. The selection of this project for study is motivated by its unique characteristics. (1) The magnitude of the engineering quantities and the corresponding substantial financial investment accentuate the necessity for the most appropriate construction approach. Furthermore, the methodology should be amenable to iterative enhancements and validation through recurrent applications. (2) Intricate geological conditions are prevalent. The engineering site is covered by a geological layer of sand with a thickness ranging from 4 to 40 meters. The majority of the pile foundations are concentrated in karst regions, constituting 54.9% of the total. (3) Karst characteristics display a diverse range of features, characterized by the existence of caves with varying vertical extents, spanning from 1 to 10 meters in height.

Representative foundation piles within this project have been selected. Taking Pile 94–4# of the project as an example, the geotechnical investigation indicates the presence of karst caves with depths of 8.7 meters and 7.7 meters, and a sand layer thickness of 37.7 meters. Based on this information, three alternative schemes [20,21,24] are proposed as shown in **Table 6**.

**Table 6:** Pile foundation construction options.

Number	Construction measures of karst cave	Advantage	Disadvantage
1	The steel casing is buried to a depth of 10 meters below the original ground level. When grouting leakage occurs, a large amount of crushed rock and clay is immediately poured into the cavity to fill it.	To ensure the stability of borehole without collapsing and relatively low construction costs.	It has a high risk of collapse, potential safety hazards, a long construction duration and a low pile formation rate.
2	The double-layer steel casing method is adopted, with the large-diameter steel casing reaching the bottom of the sand layer. When grouting leakage occurs, a large amount of crushed rock and clay is immediately poured into the cavity until the leakage stops. Then, a small-diameter steel casing is inserted.	It offers geological stability, low risk of collapse, short construction period, and high pile quality.”	The material costs are relatively high, and it requires advanced construction techniques.
3	The steel casing is buried to the bottom of the sand layer. When grouting leakage occurs, a large amount of crushed rock and clay is immediately poured into the cavity to fill it.	It facilitates material savings, reduces the probability of sand layer collapse, thereby ensuring worker safety.	It has a long construction period, significant material consumption, and average pile quality.

### 4.2 Calculation of subjective weights based on the improved ANP

Experts were invited to engage in the research questions through interviews and questionnaire completion, demographic characteristics of experts as shown in **Table 7**. Particularly, with due consideration for the domain-specific expertise of experts across different fields, the foremost five experts listed in the table were solicited to undertake the assessment of criteria, while the last five experts from the table were engaged for the selection of scheme decision, aiming to enhance the professionalism and rigor of the evaluation outcomes.

**Table 7:** Demographic characteristics of experts in this study.

	Title	Company	Education property	Working seniority	Field of Study
1	Chief Engineer	Construction part	Bachelor’s degree in Engineering	15 years	Construction technology of civil engineering
2	Department minister	Construction part		10 years	
3	Project Manager	Senior Engineer		20 years	
4	Project Manager	Owner part		20 years	
5	Chief Engineer	Design part		10 years	Architectural Design
6	Professor	University	Doctor of engineering	31 years	Road and bridge construction
7	Associate professor			21 years	Civil engineering management
8	Professor			28 years	

**Fig 4:**
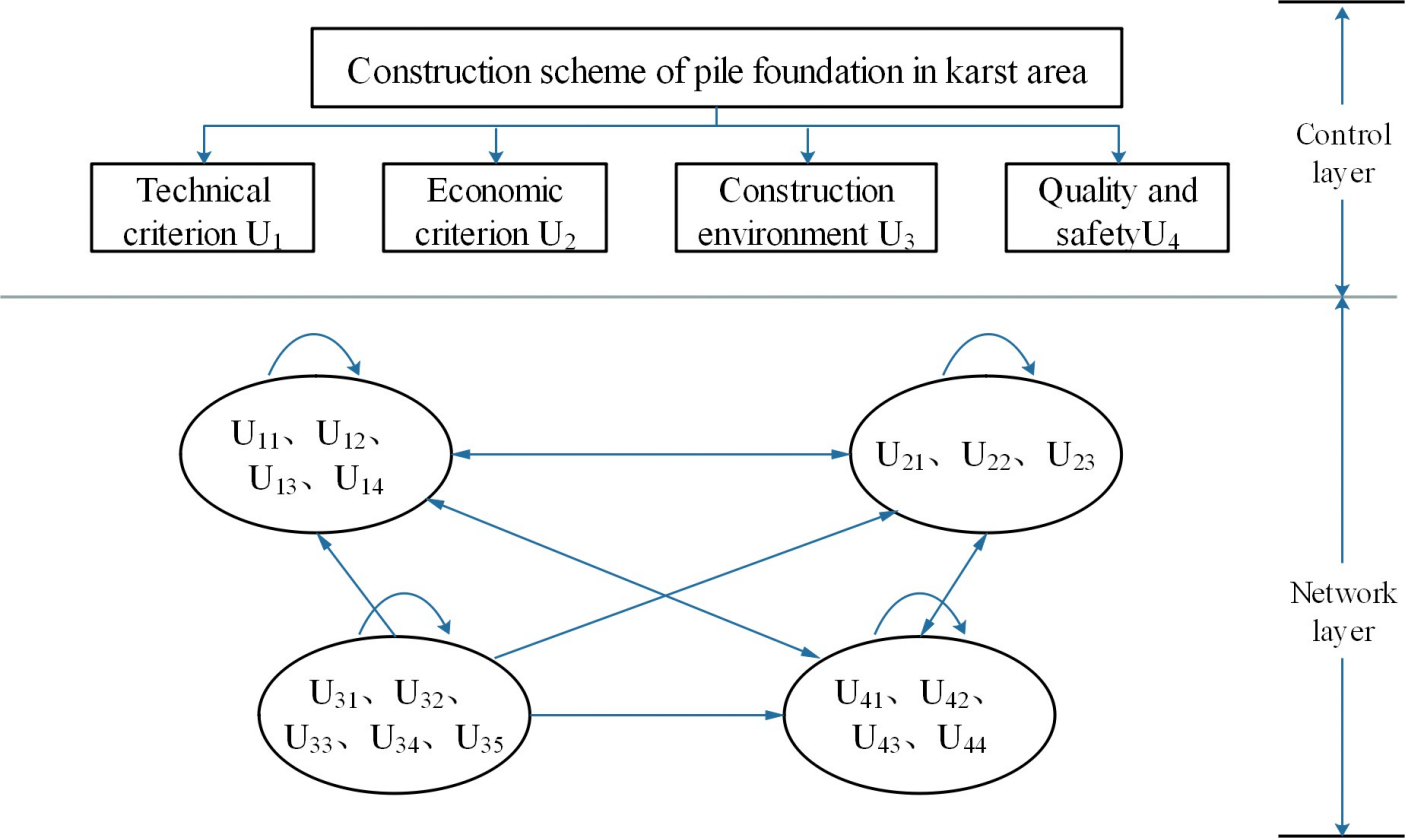
Diagram of ANP network structure.

**Fig 5:**
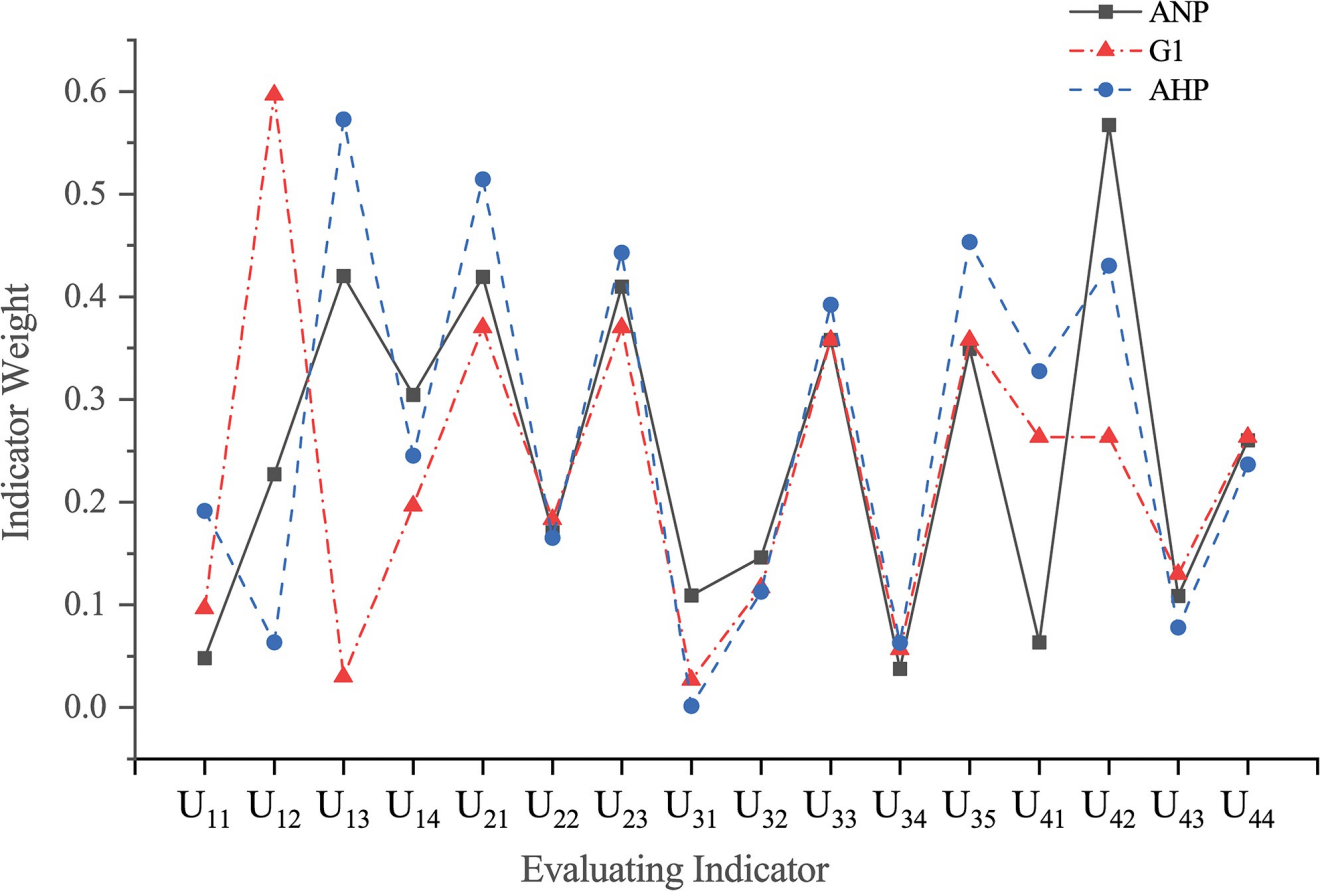
Comparison of three methods to calculate weight values.

Subsequently, based on the correlation analysis of the indicators, the establishment of a ANP network hierarchical structure was carried out, as shown in **Fig 4.** Using the secondary indicators in the technical criteria as an example, questionnaires were collected to obtain ratings from five experts for different indicators, as shown in **Table 8**. The judgment matrix was computed according to the method described in Section 2.2, and its corresponding consistency ratio (CR) was calculated using the MATLAB software, as shown in **Tables 9**–**13**. Furthermore, subjective weights were calculated using AHP and G1 methods and compared with those derived from the improved ANP method, as shown in **Fig 5.**

**Table 8:** Expert scoring values of criterion layer U_1_.

Expert	$\Delta r_{j_{1}j_{2}}$			$a_{ij_{k}}$		
1	r_34_	r_41_	r_12_	a_12_	a_23_	a_34_
	2.5	2	1.5	1.5	-6	2.5
2	r_34_	r_41_	r_12_	1.5	-5.5	2
	2	2	1.5			
3	r_31_	r_14_	r_42_	3.5	-5	3
	1.5	1.5	2			
4	r_31_	r_14_	r_42_	3	-5	3.5
	2	1.5	1.5			
5	r_43_	r_31_	r_12_	2	-3.5	-2
	2	1.5	2			
Average value				2.3	-5	1.8
Rounded number				2	-5	2

**Table 9:** Judgment matrix of criterion layer U_1_.

U_1_	1	2	3	4
1		2	1/3	1
2	1/2		1/5	1/3
3	3	5		2
4	1	3	1/2	
CR_1_ = 0.0093				

**Table 10:** Judgment matrix of criterion layer U_2_.

U_2_	1	2	3
1		3	1
2	1/3		1/2
3	1	2	
CR_2_ = 0.0176			

**Table 11:** Judgment matrix of criterion layer U_3_.

U_3_	1	2	3	4	5
1		1/4	1/7	1/2	1/8
2	4		1/3	2	1/4
3	7	3		5	1
4	2	1/2	1/2		1/6
5	8	4	1	6	
CR_3_ = 0.0118					

**Table 12:** Judgment matrix of criterion layer U_4_.

U_4_	1	2	3	4
1		1	3	1
2	1		4	2
3	1/3	1/4		1/2
4	1	1/2	1/2	
CR_4_ = 0.0172				

**Table 13:** Judgment matrix of control layer U.

U	1	2	3	4
1		2	3	2
2	1/2		1	1
3	1/3	1		1
4	1/2	1/2	1	
CR = 0.0077				

After calculation, it was found that the average CR of the judgment matrix was less than 0.1, indicating that the consistency test has passed. The subjective weights $\omega_{Subjective}{}^{U_{i}}$ of the control layer indicators can be computed using the Super Decision software.


$$\begin{array}{l} \omega_{{}_{Subjective}}{}^{U_{1}}=[\begin{array}{cccc} 0.0479 & 0.2272 & 0.4204 & 0.3045 \end{array}]\\ \omega_{{}_{Subjective}}{}^{U_{2}}=[\begin{array}{ccc} 0.4193 & 0.1706 & 0.4101 \end{array}]\\ \omega_{{}_{Subjective}}{}^{U_{3}}=[\begin{array}{ccccc} 0.1091 & 0.1461 & 0.3581 & 0.0376 & 0.3492 \end{array}]\\ \omega_{{}_{Subjective}}{}^{U_{4}}=[\begin{array}{cccc} 0.0634 & 0.5677 & 0.1088 & 0.2603 \end{array}]\\ \omega_{{}_{Subjective}}{}^{U}=[\begin{array}{cccc} 0.4336 & 0.1948 & 0.1768 & 0.1948 \end{array}] \end{array}$$

According to the analysis of expert subjective evaluations, it can be observed that in the technical criteria, time limit of construction and the difficulty of construction are relatively important. In the economic criteria, the construction cost of karst cave and the construction cost are considered significant. Regarding the operational and environmental criteria, attention should be given to the filling degree of karst caves and the maximum height of karst cavity. In terms of safety and quality criteria, the focus should be on the construction technology control measures and the emergency response plans. Among the four control criteria, experts believe that the technical criteria occupy a crucial position.

### 4.3 Computation of combined weights

Universal weights, predicted through the multiple linear regression method based on sample data, are used as objective weights. By combining the subjective weights determined through the improved Analytic Network Process (ANP) to obtain the combined weights *ω*_*Combined*_. Considering the limited sample size and the non-replicability of each engineering project, a weighted average approach is employed to determine the combined weights of the control layer indicators. This approach takes into account the inadequacy of data samples and the unique characteristics of each project, with the mean value serving as the composite weight for the control layer indicators.


$$\omega_{Combined}=[\begin{array}{cccc} 0.2185 & 0.3610 & 0.1763 & 0.2428 \end{array}]$$

When the subjective evaluation concludes that the technical criteria hold a significant position, in this case, further statistical analysis and data mining can be conducted. It becomes evident that in the decision-making process regarding pile foundation construction plans in karst areas, the technical, quality and safety, and economic criteria all possess undeniable importance that cannot be overlooked.

### 4.4 Grey-fuzzy evaluation method

#### 4.4.1 Categorizations of indicator evaluation levels

Drawing upon the existing literature and the practical engineering context, the classification of indicator evaluation levels was established. Regarding quantitative indicators, taking thickness of sand layer(U_31_) as an example, the interval partition sets for Ruan [50] are {5, 10, 15, 20}, for Huang [63] {20, 30; 40}, and Hao [57] divides 5 levels into with 3 intervals {10, 30}. Clearly, there is a lack of uniformity in the delineation ranges for the same indicator across different studies, which is in accordance with the variations in practical engineering scenarios. There are notable disparities in karst geological conditions across different regions. For instance, indicators like thickness of the first rock layer(U_32_) allow for a graded classification by calculating mechanical parameters. Conversely, metrics such as "thickness of sand layer(U_31_), the maximum height of karst cavity(U_35_), and time limit of construction(U_13_) prove challenging to generalize, given their strong contextual relevance to specific engineering conditions. Therefore, the K-Means algorithm is suggested for data clustering based on the project’s unique geological survey information. Subsequently, the results are compared with established standards in existing literature to effectively determine the classification of indicator evaluation levels.

**Fig 6:**
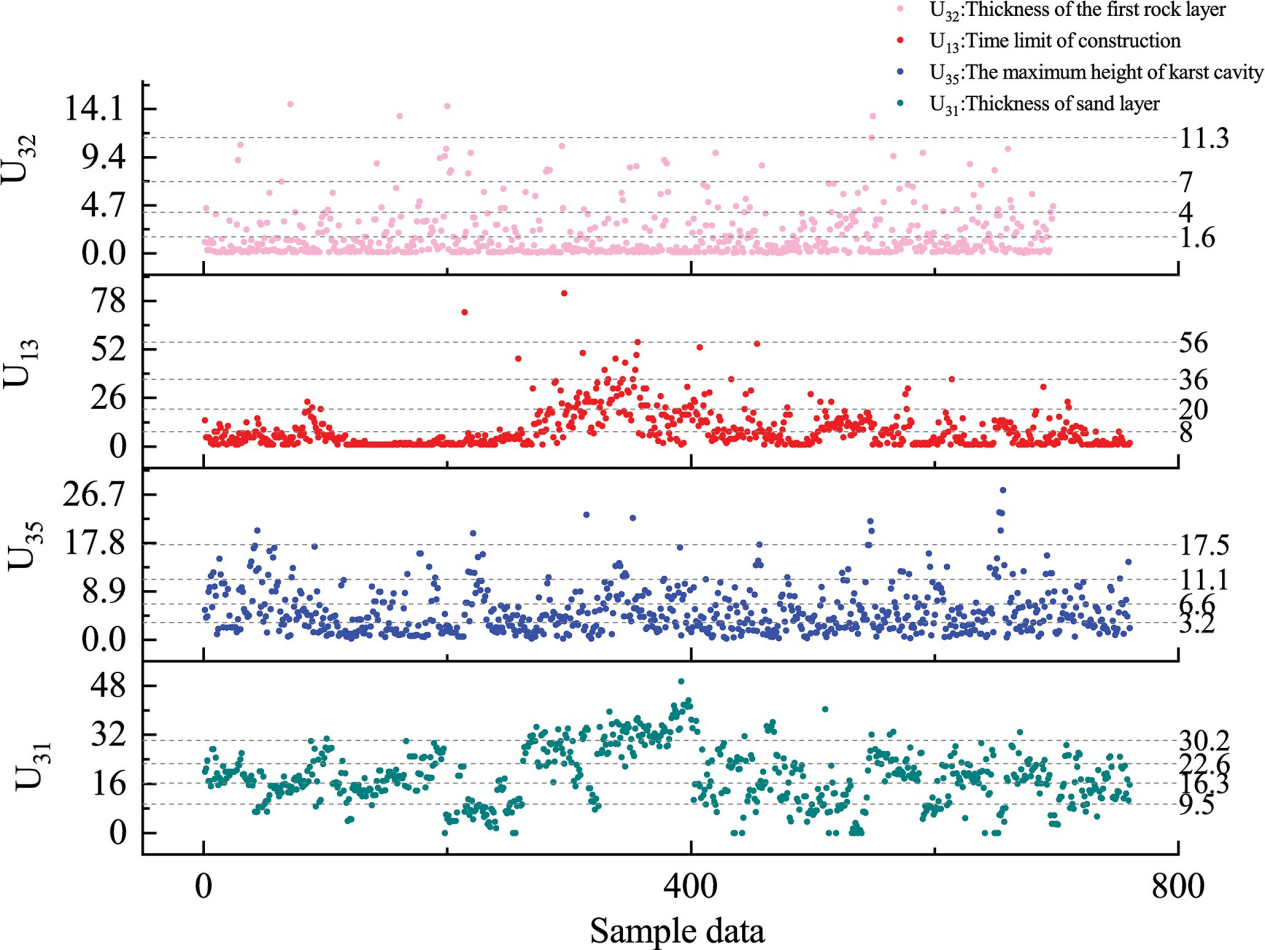
Sample data of K-means clustering.

The provided pile foundation geological survey maps were taken as the sample set, with the Euclidean distance used as a metric to measure the similarity between data objects. Based on the evaluation requirements, a total of 760 pile foundation data were selected as samples and input into the SPSS software for analysis and calculations. Firstly, the number of clusters is set to 5. Subsequently, an iterative algorithm continuously updates the positions of cluster centers by minimizing the square sum of errors within each cluster, until the objective function converges, indicating the completion of the clustering process. Finally, the clustering result is used to assign levels to the quantitative indicators. The sample data is shown in **Fig 6**, and the classification range of the secondary index is shown in **Table 14**.

**Table 14:** Category range of secondary indicators.

Control layer	Network layer	Ⅰ	Ⅱ	Ⅲ	Ⅳ	Ⅴ
U_1_	U_11_	Very good	Good	Average	Bad	Very bad
	U_12_					
	U_13_	[1,8]	[9,20]	[21,36]	[41,56]	[72,78]
	U_14_	Very Low	Low	Average	High	Very high
U_2_	U_21_	Very cheap	Cheap	Average	Expensive	Very expensive
	U_22_					
	U_23_					
U_3_	U_31_	[0–9.5]	[9.8,16.3]	[16.4,22.6]	[22.9–30.2]	[30.2–49.4]
	U_32_	[0.05–1.6]	[1.7–4]	[4.1–7]	[7.8–11.3]	[13.4–14.6]
	U_33_	Completely filled	Partially filled	Semi-filled	Few filled	Empty
	U_34_	Extremely weathering	Moderately weathered	General weathering	Micro-weathering	Unweathered
	U_35_	[0.19–3.2]	[3.3–6.6]	[6.7–11.1]	[11.2,17.5]	[19.6–27.5]
U_4_	U_41_	Very good	good	Average	Bad	Very bad
	U_42_					
	U_43_					
	U_44_					

#### 4.4.2 Construction of evaluation matrix

The data from the survey questionnaire were analyzed, and the average rating scores for each alternative solution are presented in **Table 15**. The data were incorporated into a whitenization weighting function to calculate the degree of membership of the rating values for different levels. Furthermore, employing MADM analytical approaches including cloud models, ELECTRE-II, and VIKOR methodologies. The computational processes are presented in supporting information.

**Table 15:** Evaluation matrix.

D_ij_	U_11_	U_12_	U_13_	U_14_	U_21_	U_22_	U_23_	U_31_	U_32_	U_33_	U_34_	U_35_	U_41_	U_42_	U_43_	U_44_
1	79.8	69.6	86	75.4	62.6	65	70.4	58	86	66.6	66.8	74.8	78.4	70.8	76.8	67.2
2	78.8	79.2	85.2	82.4	67.6	83.6	70.4	53.6	85.4	65	67.6	75.4	73.6	71.2	78.4	70.4
3	80	70.8	85.4	76	68.6	67	70.6	54	84.8	65	65	75.6	77.4	71.6	78.6	68.8


$$\begin{array}{l} b_{1}=\left[\begin{array}{ccccc} 0.252 & 0.380 & 0.359 & 0.009 & 0.000\\ 0.000 & 0.016 & 0.588 & 0.396 & 0.000\\ 0.088 & 0.058 & 0.262 & 0.221 & 0.176\\ 0.000 & 0.173 & 0.754 & 0.073 & 0.000 \end{array}\right]\\ b_{2}=\left[\begin{array}{ccccc} 0.292 & 0.684 & 0.024 & 0.000 & 0.000\\ 0.061 & 0.126 & 0.712 & 0.101 & 0.000\\ 0.079 & 0.067 & 0.208 & 0.227 & 0.070\\ 0.000 & 0.193 & 0.807 & 0.000 & 0.000 \end{array}\right]\\ b_{3}=\left[\begin{array}{ccccc} 0.227 & 0.442 & 0.331 & 0.000 & 0.000\\ 0.000 & 0.025 & 0.866 & 0.110 & 0.000\\ 0.070 & 0.076 & 0.198 & 0.241 & 0.065\\ 0.000 & 0.231 & 0.737 & 0.031 & 0.000 \end{array}\right] \end{array}$$

The comprehensive evaluation set was calculated based on Formula (10). The specific data are presented as follows:

$$\begin{array}{l} B_{1}=[\begin{array}{ccccc} 0.071 & 0.141 & 0.520 & 0.202 & 0.004 \end{array}]\\ B_{2}=[\begin{array}{ccccc} 0.100 & 0.254 & 0.495 & 0.076 & 0.012 \end{array}]\\ B_{3}=[\begin{array}{ccccc} 0.062 & 0.175 & 0.599 & 0.090 & 0.012 \end{array}] \end{array}$$

## 5 Evaluation result and discussion

**Fig 7:**
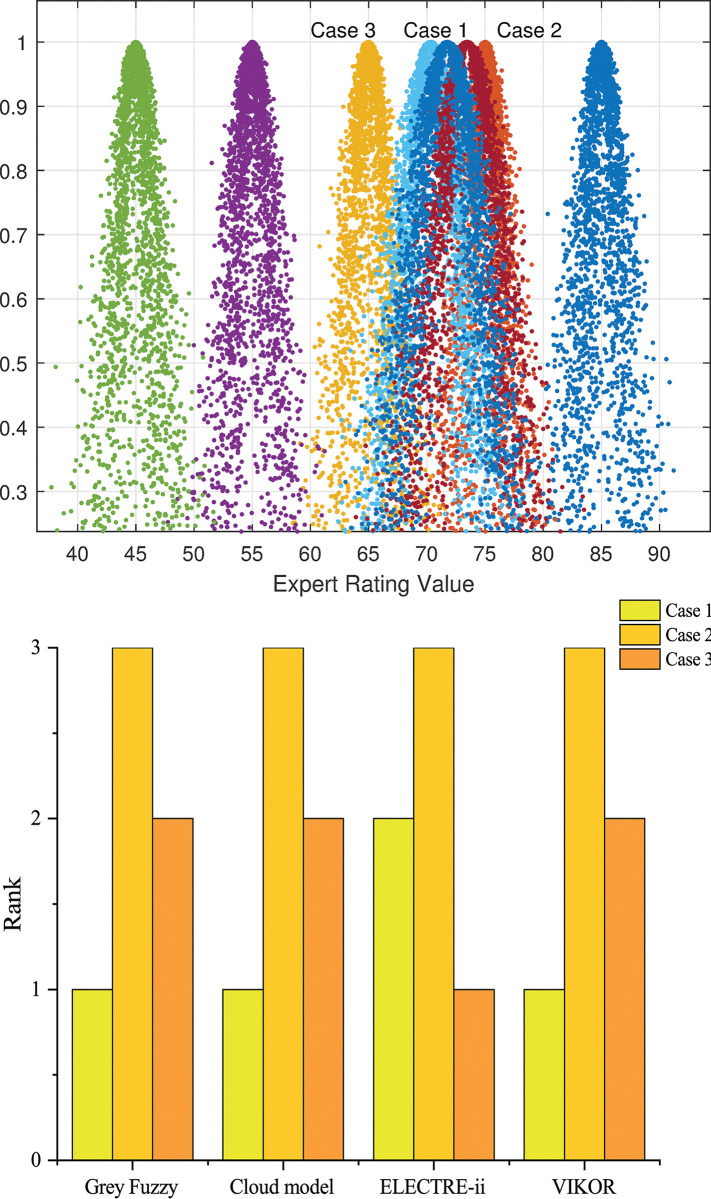
(A) Result of cloud model; (B) Comparison of results.

Based on Formula (14), the evaluation scores for each scheme were computed. Different methods’ results are presented in **Table 16**. The scheme rankings computed through various MADM methods are illustrated in **Fig 7**.

**Table 16:** Comparison of calculation results.

Scheme	Grey-Fuzzy evaluation model	Cloud Model	ELECTRE-Ⅱ	VIKOR
1	71.01	(70.34,2.84,2.72)	2	3
2	73.08	(73.47,3.21,3.17)	1	1
3	72.04	(71.76,3.16,2.66)	3	2

The effectiveness of this model is verified through comparative analysis with results obtained from other multi-attribute decision-making methods. A comparative assessment is conducted between the weight calculation employed in AHP and the G1 method. Simultaneously, supplementary validation of the comprehensive decision results is performed using the cloud model, ELECTRE-II, and VIKOR methods.

Firstly, with respect to the calculation of the evaluation indicator weights, it can be seen from **Fig 5**, the results obtained from the three methods are in substantial agreement. Nevertheless, disparities are evident between U_12_, U_13_, U_41_, and U_42_ in comparison to the remaining indicators. These discrepancies stem from the limitations inherent in conventional methodologies. Within the U_1_ layer, the weights of sequence of construction process(U_12_) and time limit of construction(U_13_), calculated using the enhanced ANP method, exhibit an inverse trend compared to other weight calculation methods. This can be attributed to the influence exerted by the construction environment(U_3_) and quality and safety(U_4_) indicators on U13, coupled with the reciprocally impactful relationship between U_13_, and the economic criterion(U_2_) indicators, consequently rendering it the highest weighted indicator in the U1 layer. Similarly, within the U4 layer, the construction technology control measures (U_42_) indicator exhibits the highest weight. Construction management system(U_41_) is primarily associated with the delineation of organizational structure and the allocation of safety responsibilities, and also represents an essential aspect in the construction of a comprehensive evaluation system. Its importance is comparatively minor. On the other hand, certain less-weighted indicators also result from the divergent assessments of the same one by multiple experts engaged in group decision-making. The improved ANP mitigates the inherent limitation in AHP and the G1 method, facilitating a comprehensive assessment of the interrelationships among indicators. Consistent with result of other methods, this validation confirms the viability and practical utility of this approach.

Additionally, the study introduces three alternative construction schemes, which primarily focused on distinct karst treatment methods. As shown in Table 16, following analysis through four distinct computational methodologies, Scheme 2 consistently exhibits superior performance, making it the optimal decision for pile foundation construction in karst area. The results of cloud model, ELECTRE-II, VIKOR, and the grey fuzzy comprehensive evaluation model methods are compared and ranked, as shown in **Fig 7B**. The consistent outcomes validate the efficacy of the methodological approach employed in this study.

The distinctive strength of cloud model resides in its capacity to effectuate the conversion of qualitative concepts into quantitative data, visually representing the grade cloud diagram, as illustrated in **Fig 7A**. ELECTRE-II and VIKOR methodologies evaluate alternative schemes in a ranked fashion, yet ELECTRE-Ⅱ is sensitive to threshold settings, leading to differing conclusions. Both methods are suitable for situations with numerous alternative options. Nevertheless, the grey fuzzy comprehensive evaluation model excels in handling uncertain information, mitigating stochasticity in membership functions, and delivering an overall integrated score. It is particularly well-suited for multi-attribute decision problems with minor score disparities and a limited number of alternative options.

During the actual construction process, there were no occurrences of hole collapse, and ultrasonic testing confirmed that it is a Class I pile, indicating that the quality requirements have been met. This demonstrates the necessity of integrated decision-making involving multiple options and suggests that the model can serve as a reference for similar projects.

## 6 Conclusions

Given the project characteristics of bridge pile foundation construction in karst areas, the selection of construction schemes is considered as a multi-attribute decision-making problem. The construction of bridge foundations is closely related to the overall progress of the project, and decision-making regarding the choice of schemes is a crucial step in the construction process. Therefore, research in this area is necessary. The major findings of this study were summarized as follows.

1. Based on the indicator of technology, economy, construction environment, and quality and safety, a decision evaluation system for the construction scheme of bridge pile foundations in karst areas has been established. The system considers the key controls of all construction schemes, making the evaluation more comprehensive and systematic. It is applicable to the assessment of most construction projects and holds significant implications for further decision-making.
2. Multiple linear regression analyzes the correlation coefficients of sample data, achieving the prediction of weights for research on similar project types. The combination of universal weights and subjective weights through weighting maximizes the objectivity of indicator weights.
3. The improved ANP enhances the consistency and effectiveness of expert assessments, reducing the practical implementation obstacles in group decision-making. Compared with the results of the AHP and G1 methodologies, the improved ANP provides more accurate weights for complex evaluation systems with interconnected indicators.
4. Given the uncertainties in certain data and the ambiguity in evaluations during underground foundation construction, the grey-fuzzy comprehensive evaluation method proves suitable for decision-making, consistently aligning with the outcomes of cloud model, ELECTRE, and VIKOR methods. The multi-attribute decision model can take into account multiple objectives in pile foundation construction in karst areas, avoiding the one-sidedness of single-factor decisions and the potential decision-making errors caused by subjective cognitive biases. It enables a scientific, comprehensive, and accurate judgment. This model serves as a valuable reference for multi-attribute decision-making in similar engineering construction projects.

Despite all the advantages, the proposed MADM model has some drawbacks that suggest directions for future research. First, while the new model addresses the integration challenges associated with expert group decision-making, the reliability of decision opinions remains influenced by the professionals’ backgrounds and vested interests. Hence, in future research, it is imperative to develop methods for determining decision-makers’ weights, as suggested by [99]. Second, the combination of universal weights and subjective weights can be achieved through alternative methods, such as the Minimum Discriminant Information method [37] and others. Thirdly, with an augmented dataset, one could explore dynamic weighting using advanced techniques like Artificial Neural Networks (ANN) [75].

## Supporting information

S1 Dataset Datasets of computational processes. This document provides detailed information on the computation processes of various methods. (https://doi.org/10.6084/m9.figshare.24476317.v1).
(XLSX)

S1 File Code of methodology. (https://doi.org/10.6084/m9.figshare.24476317.v1).
(DOCX)
