# Supplementary material for: Research on programmatic multi-attribute decision-making problem: An example of bridge pile foundation project in karst area
Source: PLoS One. 2023 Dec 4;18(12):e0295296. doi: 10.1371/journal.pone.0295296 (PMC10695384; doi:10.1371/journal.pone.0295296)
Supplement: S1 File — (https://doi.org/10.6084/m9.figshare.24476317.v1). (DOCX) [file pone.0295296.s002.docx]

**Code of consistency**

disp('input A')

A=input('A=');

[n,n] = size(A);

[V,D] = eig(A);

Max_eig = max(max(D));

[r,c]=find(D == Max_eig , 1);

CI = (Max_eig - n) / (n-1);

RI=[0 0.0001 0.52 0.89 1.12 1.26 1.36 1.41 1.46 1.49 1.52 1.54 1.56 1.58 1.59];

CR=CI/RI(n);

disp('CI=');disp(CI);

disp(' CR=');disp(CR);

if CR<0.10

disp('IF CR<0.1, A satisfies consistency!');

else

disp(' IF CR>=0.1, A needs to be modified!!');

end

**Code of AHP**

clc;clear

load A.mat

[n,n] = size(A);

[V,D] = eig(A);

Max_eig = max(max(D));

[r,c]=find(D == Max_eig , 1);

disp(' The result of calculating the weight by the eigenvalue method is:');

disp( V(:,c) ./ sum(V(:,c)) )

CI = (Max_eig - n) / (n-1);

RI=[0 0.0001 0.52 0.89 1.12 1.26 1.36 1.41 1.46 1.49 1.52 1.54 1.56 1.58 1.59];

CR=CI/RI(n);

disp('CI=');disp(CI);

disp(' CR=');disp(CR);

if CR<0.10

disp('IF CR<0.1, A satisfies consistency!');

else

disp(' IF CR>=0.1, A needs to be modified!!');

end

**Code of G1**

clear

clc

u = input(' Please rank the indicators in order of importance:');

m = size(u,2);

fprintf(' Indicator importance order is:\n')

fprintf('u(1)\n')

for i = 2 : m

fprintf(' > %s', u(i))

end

fprintf('\n\n')

for k = 1 : m-1

fprintf(' Please enter the importance ratio r_%d' between %s and %s', u(k), u(k+1), k+1)

r(k) = input(':');

fprintf(' \n The assignment results of the degree of importance rk are as follows: \n')

for k = 1 : m-1

fprintf('r_%d = w_%s/w_%s = %7.4f;\n', k+1 ,u(k), u(k+1), r(k))

end

rr = cumprod(r,'reverse');

w(m) = 1 / (1+sum(rr));

for m = m : -1 : 2

w(m-1) = w(m) * r(m-1);

end

m = size(u,2);

fprintf(' '\n The weight calculation results are as follows: \n')

for k = m : -1 : 1

fprintf('w_%s = %7.4f;\n', u(k), w(k))

end

fprintf('\n*****end*****\n'

)

%input[1,2,3,4]

%W=[w4,w3,w2,w1]

**Code of VIKOR**

clc;clear;

load Origin_Matrix;

Size_Origin_Matrix=size(Origin_Matrix);

Origin_Matrix_Row=Size_Origin_Matrix(1);

Origin_Matrix_Column=Size_Origin_Matrix(2);

Standard_Matrix=Origin_Matrix;

for i=[1:Origin_Matrix_Column]

Standard_Matrix(:,i)=(Origin_Matrix(:,i)-min(Origin_Matrix(:,i)))/...

(max(Origin_Matrix(:,i))-min(Origin_Matrix(:,i)));

end

for i=[1:Origin_Matrix_Column]

Standard_Matrix(:,i)=(max(Origin_Matrix(:,i))-Origin_Matrix(:,i))/...

(max(Origin_Matrix(:,i))-min(Origin_Matrix(:,i)));

end

display(Standard_Matrix)

load Omega;

Best_value=[ ];

Worst_value=[ ];

for j=[1:Origin_Matrix_Column]

Best_value(end+1)=max(Standard_Matrix(:,j));

Worst_value(end+1)=min(Standard_Matrix(:,j));

end

s_Token=[ ]; % s are parts of population utility value.

for j=[1:Origin_Matrix_Column]

for i=[1:Origin_Matrix_Row]

s_Token(end+1)=Omega(j)*(Best_value(j)-Standard_Matrix(i,j))./(Best_value(j)-Worst_value(j));

end

end

s=[ ];

for i=[1:Origin_Matrix_Row]

row=[s_Token(i),s_Token(i+Origin_Matrix_Column)];

s=[s;row];

end

display(s)

S=[ ]; % S is population utility value.

for i=[1:Origin_Matrix_Row]

S(end+1)=[sum(s(i,:))];

end

display(S)

%ÇóR

R=[ ];

% R is individual regret value.

for i=[1:Origin_Matrix_Row]

R(end+1)=[max(s(i,:))];

end

display(R)

Best_S=min(S);

Worst_S=max(S);

Best_R=min(R);

Worst_R=max(R);

v=0.5% v is decision mechanism coefficient.

Q=[ ];% Q is the evaluation index.

for i=[1:Origin_Matrix_Row]

Q(end+1)=v*(S(i)-Best_S)/(Worst_S-Best_S)+(1-v)*(R(i)-Best_R)/(Worst_R-Best_R);

end

display(Q)

Result_1='The best plan is A';

Result_2=' and';

Result_3=' A';

Result_4='.';

Q_1st=min(Q)

Q_2nd=min(Q(find(Q-min(Q))))

if S(find(Q==Q_1st))<S(find(Q==Q_2nd))

if R(find(Q==Q_1st))<R(find(Q==Q_2nd))

disp('Condition 2 is satisfied.')

Result=[Result_1,num2str(find(Q==min(Q))),Result_4];

end

elseif (Q_2nd-Q_1st)>(1/(length(Q)-1))

disp('Condition 2 is not satisfied.')

disp('Condition 1 is not satisfied.')

Result=[Result_1,num2str(find(Q==min(Q))),Result_2,Result_3,...

num2str(find(Q==min(Q(find(Q-min(Q)))))),Result_4];

else disp('Condition 2 is not satisfied.')

disp('Condition 1 is satisfied.')

Result=[Result_1,num2str(find(Q==min(Q))),Result_4];

end

disp(Result)

**Code of ELECTRE-I (Python)**

**(https://github.com/Valdecy/J-Electre.git)**

# Module Created by: Prof. Valdecy Pereira, D.Sc.

# UFF - Universidade Federal Fluminense (Brazil)

# email: valdecy.pereira@gmail.com

# GitHub Repository: <https://github.com/Valdecy>

import math

import matplotlib.pyplot as plt

import numpy as np

import sys

from collections import defaultdict

# Function: Cycle Finder

# (autor:Luke Harold Miles, code at https://gist.github.com/qpwo/272df112928391b2c83a3b67732a5c25)

def simple_cycles(G):

def _unblock(thisnode, blocked, B):

stack = set([thisnode])

while stack:

node = stack.pop()

if node in blocked:

blocked.remove(node)

stack.update(B[node])

B[node].clear()

G = {v: set(nbrs) for (v,nbrs) in G.items()}

sccs = strongly_connected_components(G)

while sccs:

scc = sccs.pop()

startnode = scc.pop()

path = [startnode]

blocked = set()

closed = set()

blocked.add(startnode)

B = defaultdict(set)

stack = [ (startnode,list(G[startnode])) ]

while stack:

thisnode, nbrs = stack[-1]

if nbrs:

nextnode = nbrs.pop()

if nextnode == startnode:

yield path[:]

closed.update(path)

elif nextnode not in blocked:

path.append(nextnode)

stack.append( (nextnode, list(G[nextnode])) )

closed.discard(nextnode)

blocked.add(nextnode)

continue

if not nbrs:

if thisnode in closed:

_unblock(thisnode, blocked, B)

else:

for nbr in G[thisnode]:

if thisnode not in B[nbr]:

B[nbr].add(thisnode)

stack.pop()

path.pop()

remove_node(G, startnode)

H = subgraph(G, set(scc))

sccs.extend(strongly_connected_components(H))

# Function: SCC

def strongly_connected_components(graph):

index_counter = [0]

stack = []

lowlink = {}

index = {}

result = []

def _strong_connect(node):

index[node] = index_counter[0]

lowlink[node] = index_counter[0]

index_counter[0] += 1

stack.append(node)

successors = graph[node]

for successor in successors:

if successor not in index:

_strong_connect(successor)

lowlink[node] = min(lowlink[node],lowlink[successor])

elif successor in stack:

lowlink[node] = min(lowlink[node],index[successor])

if lowlink[node] == index[node]:

connected_component = []

while True:

successor = stack.pop()

connected_component.append(successor)

if successor == node: break

result.append(connected_component[:])

for node in graph:

if node not in index:

_strong_connect(node)

return result

# Function: Remove Node

def remove_node(G, target):

del G[target]

for nbrs in G.values():

nbrs.discard(target)

# Function: Subgraph

def subgraph(G, vertices):

return {v: G[v] & vertices for v in vertices}

###############################################################################

# Function: Concordance Matrix

def concordance_matrix(dataset, W):

concordance = np.zeros((dataset.shape[0], dataset.shape[0]))

for i in range(0, concordance.shape[0]):

for j in range(0, concordance.shape[1]):

value = 0

for k in range(0, dataset.shape[1]):

if (dataset[i,k] >= dataset[j,k]):

value = value + W[k]

concordance[i,j] = value

if (np.sum(W) != 0):

concordance = concordance/np.sum(W)

return concordance

# Function: Discordance Matrix

def discordance_matrix(dataset):

delta = np.max(np.amax(dataset, axis = 0) - np.amin(dataset, axis = 0))

discordance = np.zeros((dataset.shape[0], dataset.shape[0]))

for i in range(0, discordance.shape[0]):

for j in range(0, discordance.shape[1]):

discordance[i,j] = np.max((dataset[j,:] - dataset[i,:]))/delta

if (discordance[i,j] < 0):

discordance[i,j] = 0

return discordance

# Function: Dominance Matrix

def dominance_matrix(concordance, discordance, c_hat = 0.75, d_hat = 0.50):

dominance = np.zeros((concordance.shape[0], concordance.shape[0]))

for i in range (0, dominance.shape[0]):

for j in range (0, dominance.shape[1]):

if (concordance[i,j] >= c_hat and discordance[i,j] <= d_hat and i != j):

dominance[i, j] = 1

return dominance

# Function: Find Cycles and Unites it as a Single Criteria

def johnson_algorithm_cycles(dominance):

graph = {}

value = [[] for i in range(dominance.shape[0])]

keys = range(dominance.shape[0])

for i in range(0, dominance.shape[0]):

for j in range(0, dominance.shape[0]):

if (dominance[i,j] == 1):

value[i].append(j)

for i in keys:

graph[i] = value[i]

s1 = list(simple_cycles(graph))

for k in range(0, len(s1)):

for j in range(0, len(s1[k]) -1):

dominance[s1[k][j], s1[k][j+1]] = 0

dominance[s1[k][j+1], s1[k][j]] = 0

s2 = s1[:]

for m in s1:

for n in s1:

if set(m).issubset(set(n)) and m != n:

s2.remove(m)

break

for i in range(0, dominance.shape[0]):

count = 0

for j in range(0, len(s2[k])):

if (dominance[i, s2[k][j]] > 0):

count = count + 1

if (count > 0):

for j in range(0, len(s2[k])):

dominance[i, s2[k][j]] = 1

return dominance

# Function: Electre I

def electre_i(dataset, W, remove_cycles = False, c_hat = 0.75, d_hat = 0.50, graph = True):

kernel = []

dominated = []

concordance = concordance_matrix(dataset, W)

discordance = discordance_matrix(dataset)

dominance = dominance_matrix(concordance, discordance, c_hat = c_hat, d_hat = d_hat)

if (remove_cycles == True):

dominance = johnson_algorithm_cycles(dominance)

row_sum = np.sum(dominance, axis = 0)

kernel = np.where(row_sum == 0)[0].tolist()

for j in range(0, dominance.shape[1]):

for i in range(0, len(kernel)):

if (dominance[kernel[i], j] == 1):

if (j not in dominated):

dominated.append(j)

limit = len(kernel)

for j in range(0, dominance.shape[1]):

for i in range(0, limit):

if (dominance[kernel[i], j] == 0 and np.sum(dominance[:,j], axis = 0) > 0):

if (j not in dominated and j not in kernel):

kernel.append(j)

kernel = ['a' + str(alt + 1) for alt in kernel]

dominated = ['a' + str(alt + 1) for alt in dominated]

if (graph == True):

for i in range(0, dominance.shape[0]):

radius = 1

node_x = radius*math.cos(math.pi * 2 * i / dominance.shape[0])

node_y = radius*math.sin(math.pi * 2 * i / dominance.shape[0])

if ('a' + str(i+1) in kernel):

plt.text(node_x, node_y, 'a' + str(i+1), size = 12, ha = 'center', va = 'center', bbox = dict(boxstyle = 'round', ec = (0.0, 0.0, 0.0), fc = (0.8, 1.0, 0.8),))

else:

plt.text(node_x, node_y, 'a' + str(i+1), size = 12, ha = 'center', va = 'center', bbox = dict(boxstyle = 'round', ec = (0.0, 0.0, 0.0), fc = (1.0, 0.8, 0.8),))

for i in range(0, dominance.shape[0]):

for j in range(0, dominance.shape[1]):

node_xi = radius*math.cos(math.pi * 2 * i / dominance.shape[0])

node_yi = radius*math.sin(math.pi * 2 * i / dominance.shape[0])

node_xj = radius*math.cos(math.pi * 2 * j / dominance.shape[0])

node_yj = radius*math.sin(math.pi * 2 * j / dominance.shape[0])

if (dominance[i, j] == 1):

if ('a' + str(i+1) in kernel):

plt.arrow(node_xi, node_yi, node_xj - node_xi, node_yj - node_yi, head_width = 0.01, head_length = 0.2, overhang = 0.0, color = 'black', linewidth = 0.9, length_includes_head = True)

else:

plt.arrow(node_xi, node_yi, node_xj - node_xi, node_yj - node_yi, head_width = 0.01, head_length = 0.2, overhang = 0.0, color = 'red', linewidth = 0.9, length_includes_head = True)

axes = plt.gca()

axes.set_xlim([-radius, radius])

axes.set_ylim([-radius, radius])

plt.axis('off')

#plt.show()

return concordance, discordance, dominance, kernel, dominated,plt

###############################################################################

**Code of Cloud mode**

clc;clear;close all;

N=3000;

Ex1=85;

En1=1.6667;

He1=1;

Ex2=75;

En2=1.6667;

He2=1;

Ex3=65;

En3=1.6667;

He3=1;

Ex4=55;

En4=1.6667;

He4=1;

Ex5=45;

En5=1.6667;

He5=1;

Ex6=70.34;

En6=2.84;

He6=1;

Ex7=73.47;

En7=3.2;

He7=1;

Ex8=71.76;

En8=3.2;

He8=1;

CloudDrp=zeros(2,N);

for i=1:N

E_n1=normrnd(En1,He1,1,1);

CloudDrp(1,i)=normrnd(Ex1,E_n1,1,1);

CloudDrp(2,i)=exp(-(CloudDrp(1,i)-Ex1)^2/(2*E_n1^2));

E_n2=normrnd(En2,He2,1,1);

CloudDrp(3,i)=normrnd(Ex2,E_n2,1,1);

CloudDrp(4,i)=exp(-(CloudDrp(3,i)-Ex2)^2/(2*E_n2^2));

E_n3=normrnd(En3,He3,1,1);

CloudDrp(5,i)=normrnd(Ex3,E_n3,1,1);

CloudDrp(6,i)=exp(-(CloudDrp(5,i)-Ex3)^2/(2*E_n3^2));

E_n4=normrnd(En4,He4,1,1);

CloudDrp(7,i)=normrnd(Ex4,E_n4,1,1);

CloudDrp(8,i)=exp(-(CloudDrp(7,i)-Ex4)^2/(2*E_n4^2));

E_n5=normrnd(En5,He5,1,1);

CloudDrp(9,i)=normrnd(Ex5,E_n5,1,1);

CloudDrp(10,i)=exp(-(CloudDrp(9,i)-Ex5)^2/(2*E_n5^2));

E_n6=normrnd(En6,He6,1,1);

CloudDrp(11,i)=normrnd(Ex6,E_n6,1,1);

CloudDrp(12,i)=exp(-(CloudDrp(11,i)-Ex6)^2/(2*E_n6^2));

E_n7=normrnd(En7,He7,1,1);

CloudDrp(13,i)=normrnd(Ex7,E_n7,1,1);

CloudDrp(14,i)=exp(-(CloudDrp(13,i)-Ex7)^2/(2*E_n7^2));

E_n8=normrnd(En8,He8,1,1);

CloudDrp(15,i)=normrnd(Ex8,E_n8,1,1);

CloudDrp(16,i)=exp(-(CloudDrp(15,i)-Ex8)^2/(2*E_n8^2));

end

plot(CloudDrp(1,:),CloudDrp(2,:),'.')

hold on

plot(CloudDrp(3,:),CloudDrp(4,:),'.')

hold on

plot(CloudDrp(5,:),CloudDrp(6,:),'.')

hold on

plot(CloudDrp(7,:),CloudDrp(8,:),'.')

hold on

plot(CloudDrp(9,:),CloudDrp(10,:),'.')

plot(CloudDrp(11,:),CloudDrp(12,:),'.')

text(76,1.02,'Case 2','FontSize',10)

plot(CloudDrp(13,:),CloudDrp(14,:),'.')

text(68,1.02,'Case 1','FontSize',10)

plot(CloudDrp(15,:),CloudDrp(16,:),'.')

text(60,1.02,'Case 3','FontSize',10)

grid on

xlabel('Expert Rating Value')
